# Understanding the employment impact of neuromyelitis optica spectrum disorder in the USA: Mixed methods

**DOI:** 10.3389/fneur.2023.1142640

**Published:** 2023-03-09

**Authors:** Farrah J. Mateen, Cristina M. Trápaga Hacker

**Affiliations:** Harvard Medical School, Massachusetts General Hospital, Boston, MA, United States

**Keywords:** neuromyelitis optica (NMO), employment, unemployment, disability, outcomes, qualitative research

## Abstract

Neuromyelitis optica spectrum disorder (NMOSD) is a rare and disabling neurological disorder, marked by recurrent attacks of the central nervous system. NMO has a high female predominance and disproportionately affects racial and ethnic groups who are under- and unemployed in the USA. Three focus groups, involving 20 working age adults with NMOSD in the USA, were convened *via* Zoom online, to discuss the topic of employment in NMOSD. Consolidated Criteria for Reporting Qualitative research (COREQ) were followed. Discussions were coded for major themes using an inductive approach. The following themes emerged: (1) Barriers due to NMOSD on employment including (i) visible and invisible symptoms, (ii) the burden of treatment, and (iii) time to diagnosis; (2) Mitigating factors when NMOSD affects employment; (3) Impact of COVID-19; (4) Impact on income; (5) Impact on new and future employment and higher education opportunities; and (6) Unmet needs that are pragmatically addressable, outside of major policy or scientific changes.

## Introduction

Neuromyelitis optica spectrum disorder (NMOSD) is a rare and disabling neurological disorder, marked by recurrent attacks of the central nervous system. NMO has a high female predominance (>85%) and affects a high number of Black, Latinx, and Asian people (1–3). These racial and ethnic groups are also disproportionately affected by unemployment, under-employment, and wage loss in the USA (4). Despite a surge in NMO research in recent years, almost nothing is reported on employment, job loss, personal income, and work hours among people with NMOSD. However, given the profound nature of attacks of blindness and paralysis as well as other reported symptoms, the impact of NMOSD on employment is anecdotally thought to be high. Using a digital, cross-sectional survey of 193 people with NMOSD (5), reported in 2019, an estimated 35% of people with NMOSD were employed and 21% were employed full-time. Two-thirds of the unemployed NMOSD respondents reported being disabled (5).

In related diseases, such as multiple sclerosis (MS), reports of the socioeconomic impact on people living with the disease has led to a more complete understanding of the psychosocial toll, specific risks, unmet service needs, and remediable factors leading to opportunity loss (6–8). Similar work has yet to occur in NMOSD, perhaps given the relatively recent distinction of the condition from MS using antibody biomarkers. In general, the NMOSD patient's perspective is under-reported in the medical literature.

Given the limited understanding of NMOSD on unemployment in the USA, qualitative methods were chosen to provide formative understanding of the topic. Focus groups of people with NMOSD were convened to gather the range of issues facing employed and unemployed people with NMOSD.

## Methods

The Massachusetts General Hospital's Institutional Review Board reviewed the study protocol and deemed it exempt from further review. Participants provided their own individual verbal and written consent to participate. Inclusion criteria were a diagnosis of NMOSD by a neurologist (aquaporin-4 antibody seropositive, myelin oligodendrocyte glycoprotein (MOG) seropositive, or double seronegative) with confirmation in a medical record reviewed by a neurologist-investigator, age 18–70 years old at the time of enrollment, and residence in the USA. Exclusion criteria were an unverified diagnosis of NMOSD, residence outside of the USA, or NMOSD diagnosed < 1 month ago. Participants could speak English or have a translator available in their language of choice. A sample size of 20 participants was targeted.

Participants were recruited through the NMO clinics at Massachusetts General Hospital and through the word of mouth, social media posting to a recruitment website from the Sumaira Foundation and Massachusetts General Hospital (Rally), and *via* neurologists in other U.S. centers. Participants were recruited to achieve diversity of race, gender, ethnicity, geographic location in the USA, and autoantibody status. Participants who provided proof of their diagnosis were selected until the targeted sample size of 20 people was reached. Six people aiming to enroll in the focus groups had fabricated documentation of NMOSD upon review and were excluded from participation.

Three focus groups were convened *via* Zoom™ within an 8-day timespan in December 2022. This timing reflects the gradual taper of COVID-19 cases in the USA, particularly COVID-19-related hospitalizations and deaths. It is a high-inflation, economic slowdown environment; employment remains strong for the general U.S. population. In December 2022, unemployment was low (3.7%) in the U.S. population (9).

The interviewer was the principal investigator (female, faculty neuroimmunology-trained neurologist with a PhD in Epidemiology) and was assisted by a clinical research coordinator, fluent in English and Spanish. The participants were aware of the topic of the discussion “employment and NMO” and before the discussion, asked to complete a structured ~75-question survey in English or Spanish on employment and NMOSD to aid survey development. The survey was pilot tested by telephone with NMOSD participants before enrollment in this study. All focus groups were held at 4-5:30 PM and with notice to allow working people to participate close to the end—or after—their regular employment hours. Each participant was paid 100 USD for the focus group discussion and 75 USD for the survey completion. We chose to pay participants to ensure employed participants were represented alongside those who were un- or under-employed, all without loss of possible income during participation.

The goal of the focus group—to understand the impact of NMOSD on employment—was stated at the beginning of the open discussion.

Audio transcripts were made using Microsoft Word™ and word frequencies were noted for possible NMO symptoms (e.g., “fatigue”) and employment-related words (e.g., “part-time”). Both investigators wrote extended written notes during the discussions and coded key words post-focus groups. A thematic, inductive approach was used to organize the major issues that emerged. Prompt questions were used for the discussions, but no themes were pre-conceived. Post-focus groups, the following themes emerged: (1) Barriers due to NMOSD on employment including (i) symptoms, (ii) the burden of treatment, and (iii) time to diagnosis; (2) Mitigating factors when NMOSD affects employment; (3) Impact of COVID-19; (4) Impact on Income; (5) Impact on new and future employment and higher education opportunities; and (6) Unmet needs that are pragmatically addressable, outside of major policy or scientific changes.

Reporting of the qualitative research followed the checklist for the Consolidated Criteria for Reporting Qualitative research (COREQ) (10). Transcripts and investigators' coded notes were not sent back to the participants, and there were no repeat interviews after the focus groups, i.e., each participant contributed through a single survey and focus group encounter. The sponsor of the study was Horizon Therapeutics, a pharmaceutical company focused on rare diseases, and who manufactures a disease modifying therapy (DMT) for NMOSD. Additional support to plan the study was provided by the Sumaira Foundation for NMO. The sponsors had no role in the design, conduct, or reporting of this research or the decision to submit this research for publication.

## Results

Participants' demographic, clinical, and employment characteristics are provided in Table 1. All participants were present throughout the entire focus group. Two caregivers were also available for two participants, including one who provided translation from Spanish.

**Table 1:** Participant characteristics (*n* = 20).

**Variable**	**Count**
Age (years), mean	43.9
Age (years), range	27–62
**Sex**	***n*** **(%)**
Female	17 (85)
Male	3 (15)
**Race**	
White	9 (45)
Black or African American	8 (40)
Asian	2 (10)
Other	2 (10)
**Hispanic or Latinx**	
Yes	3 (15)
No	17 (85)
**Marital status**	
Single	2 (10)
Married/partnership	16 (80)
Divorced	2 (10)
**Highest level of completed education**	
High School/GED	9 (45)
Trade School	1 (5)
College/Bachelor's Degree	6 (30)
Professional Degree	4 (20)
Number of people living in household, mean	3.6
Number of household income earners, mean	2
**Positive for the aquaporin-4 antibody**	
Yes	15 (75)
No	5 (25)
**Positive for the myelin oligodendrocyte glycoprotein (MOG) antibody**	
Yes	2 (10)
No	18 (90)
**Employed at the time of NMOSD diagnosis**	
Yes	17 (85)
No	3 (15)
**Employment status at the time of first NMO attack**	
Self employed	4 (20)
Paid full time (40 h/week)	12 (60)
Paid part time	1 (5)
Disabled	1 (5)
Student	2 (10)
Work hours per week at/before the time of first NMO attack, mean	37.8
Age (years) at first NMO attack, mean	36.3
Age (years) at first NMOSD attack, range	10–57
Age (years) at NMOSD diagnosis, mean	38.6
Age (years) at NMOSD diagnosis, range	24–56
**Current employment status**	
Self employed	2 (10)
Paid full time (40 h/week)	8 (40)
Paid part time	2 (10)
Retired	1 (5)
Disabled	6 (30)
Student	1 (5)
Current work hours per week outside of home, mean	10.8
Work hours in the last 30 days, mean	82.5
Work hours missed because of problems associated with NMOSD, mean	13.4
NMOSD effect on productivity while working in the last 30 days [scale 0 (least)−10 (most)], mean	5.2
**Fatigue related to NMOSD**	
Yes	17 (85)
No	3 (15)
**Fatigue related to NMOSD led to reduce working hours or stop working altogether**	
Yes	13 (65)
No	7 (35)
**Pain related to NMOSD**	
Yes	17 (85)
No	3 (15)
**Pain impact ability to work or complete job duties**	
Yes	12 (60)
No	8 (40)
Annual income at/before the time of first NMO attack (US dollars), median (18 earners)	48,500
Current annual income (US dollars), median (19 earners)^*^	40,000

* Income numbers are not adjusted for inflation. NMO disease duration averaged 5 years.

### Barriers due to NMOSD on employment

All participants reported difficulties from NMOSD on their employment. The age of onset of NMOSD was relevant: in cases of pediatric or early adulthood-onset NMOSD, employment opportunities were especially hindered or never possible. By contrast, NMOSD onset later in adulthood, after educational attainment and career advancement, was often met with accommodations. Several participants reported they were not currently employed and were on disability status, retired early, or working free-lance.

Barriers to employment emerged as (i) physical/visible, (ii) physical/invisible, (iii) fatigue, (iv) mental health capacity, (v) cognitive, (vi) sense of negative perceptions of coworker and employers. Overall, spinal cord syndromes had a major impact on participants' employment and fatigue was the most commonly reported symptom impacting employment productivity.

Visual symptoms impacted employment through visual loss (i.e., blindness), double vision, and visual fatigue with reading. Significant resiliency was noted in visual function compensation including one participant who worked in information technology for more than 25 years with visual function in one eye, using magnifying glasses, climbing ladders, and carrying heavy equipment. A different participant with unilateral visual loss reported that coworkers continually forgot she could not see them out of one eye. Double vision was prohibitive for driving to work in another case. Visual fatigue or eye strain was notably difficult for students who felt they could not compete with long reading assignments or tasks that required long hours of visual attention.

Spinal cord symptoms predominated the discussions on barriers to employment in NMOSD. One participant reported that her “legs giving out and peeing myself” were major concerns for returning to the office. She could not wear a bra comfortably due to paresthesias and myelopathic pain, preventing a return to work. Frequent urination led to permanent disability in one participant who reported the urge to “urinate every 10 min.” Other participants reported the embarrassment of wearing adult diapers at work and the pain of sitting in a wheelchair all day. Spasms in the legs were a hindrance in some participants who found the episodes painful and difficult to predict and control. One participant reported needing to take a freight elevator into the office due to the lack of a passenger elevator. She expressed concern about coworkers watching her walk to her desk, and the resultant need to arrive early at the office to avoid being seen walking. Conversely, one participant reported that she could no longer go for lunch with her colleagues because the difficulty of getting there due to the inconvenience to go outside of the office with a physical disability, and over time, dwindling invitations by her coworkers to join in social activities at lunchtime.

While some NMOSD symptoms were perceived to be obvious to coworkers and employers, such as ambulation difficulties, several symptoms, including pain, spasms, and cognitive symptoms were felt to be invisible and overlooked. These impairments could compound. A participant whose first NMOSD symptom was binocular visual loss was able to attend college and a work study program, but after college, she experienced myelitis. “I got paralyzed as a quadriplegic and I lost the sensation of touch. That's what made it super difficult... trying to find a job that can work with those multiple disabilities.”

The need to “remember everything” and embarrassing levels of “forgetting” were reported by some people with NMOSD. One stated her desk was filled with sticky notes and notepads and was worried that the many reminders looked concerning to her colleagues. Several participants noted “brain fog” to a degree that was “unreal” and an important part of their inability to work.

One participant reported “we can't depend on ourselves” and was concerned about liability on the job or responsibility for things going wrong if her body could not see or walk in the same way as before the diagnosis.

The *burden of treatment* in NMOSD was high in some cases. The accrual of visits was burdensome over time with several participants reporting the need to travel long distances to get NMO specialized care. The initial hospitalization upon first NMOSD attack for one patient was 37 days, leading to an immediate long-term work absence. Frequency of infusions was a burden in some participants, including the need for intravenous immunoglobulin every 6 weeks (for hypogammaglobulinemia on B-cell depleting therapy) or the need for an infused DMT every 2 weeks (eculizumab). Post-infusion side effects of Benadryl and steroids were also noted to be physically tiring in some participants, requiring time off from work. One participant reported complications of plasmapheresis, leading to hospitalization for venous thromboses in the intensive care unit. One participant reported needing to see “15 specialists for her care” which was a full-time engagement.

Steroids were very commonly reported to create side effects that were difficult to work with including jitteriness, anxiety, emotionality, irritability, speaking too quickly, inattention, and overeating. “I would say crazy things” and “get very angry” said one participant. Another participant who took steroids for 19 weeks said that steroids were “the worst part” of treatment when trying to work. A third participant stated, “steroids are the devil.” The “emotional toll” of steroids on people with NMOSD and on caretakers was noted in many cases, with some reporting that they left a “permanent scar” and “reduced (completed) workloads by half.”

The needs to “plan everything,” “leave work early,” “switch to weekend appointments,” and to “travel 1.5 h to ERs where (health care workers) know NMO” were noted. However, this shifting of appointments to weekends and afterhours “greatly diminished free and recharge time” after work. Treatments were especially burdensome in some jobs where limited and often advance scheduling of time off was the norm, such as for schoolteachers, technicians, and police officers. The benefits of physical therapy were noted, but required two to three visits per week, during work hours, often requiring 5 h per work week. Aqua therapy was felt to be helpful, but “time consuming.” People in jobs were sensitive to “taking more time off than allowed.” One participant reported she was in an NMOSD clinical trial for a DMT, requiring regular study visits in addition to her usual clinical care.

The *long duration before the diagnosis of NMOSD* was also problematic for some participants, who were initially misdiagnosed with MS, or whose symptoms were dismissed as “hysterical” or “vitamin deficiency,” and led to years of “back and forth with neurologists” before the NMOSD diagnosis was rendered. The interplay between being African American, female, and having NMOSD was also noted in some but not all cases. Six hospital stays were required for one participant before the diagnosis was made who stated, “I had to firmly advocate for myself to get the right treatment.” Several participants were initially diagnosed with MS and told their school or employers this diagnosis initially. One woman reported being diagnosed with MS for 8 years before her NMOSD diagnosis was made. A participant called this the “triple disadvantage” for employment, encompassing gender, race, and “navigating the medical system with a rare disease.” Several African American women perceived that their pain in particular was “dismissed” by physicians. One man with NMOSD who presented atypically stated: “I went through the hell of ‘I don't know if I've got NMO' for a long time.”

Coworkers' perceptions influenced several participants in their work environments. One participant stated the need to “over-explain, over-document, and over-compensate” for her disease. The number of times to mention or bring up the diagnosis to an employer was a source of stress at times. Although most employers knew of the diagnosis of NMOSD among the participants, some participants had not disclosed the diagnosis to their coworkers. Participants reported “doing things differently” and that one “can't work as hard as I used to,” leading to lower work performance.

Major themes in work participation included (a) the unpredictability of the disease evolution, (b) the new excess burden of work given impairments from NMOSD, (c) employers' fear of employing someone with NMOSD, (d) driving to work difficulties, and (e) contemporaneous presence of other life factors, including parenthood and medical comorbidities.

Given the rarity of the disease and the data on NMOSD outcomes online, namely that there is a high risk of mortality within 5 years (11), many participants reported the uncertainty of their work performance on a day-to-day basis. Many were concerned about a future relapse, other NMOSD symptoms, or risk of infection (particularly progressive multifocal leukoencephalopathy). At times, this required counseling and cognitive behavioral therapy. One participant reported always waiting for “the other shoe to drop.” The mental health aspects of NMOSD were felt to be under-appreciated by others, particularly employers, and several remarked they had been told “you look healthy.” NMOSD was stated as “more than a CNS condition. It affects mental capacity. It's every piece of you.” In these settings of employment decision-making, “health trumps all.”

Two participants reported losing a job opportunity due to NMOSD. One participant reported that the employer heard about the NMOSD diagnosis after the interview, and “the job offer disappeared.” Another reported a relapse on the day she was meant to start a new job and was told she “wasn't a good fit.”

Driving was impacted in terms of sensory symptoms (“numbness and tingling” and feeling the pedals of the car), double vision, myelopathic pain, and episodic leg spasms. Fatigue and hypersomnia were noted as dangerous to drive for some participants. Some participants reported being heckled for using disability parking spots by members of the general public since their disease was less visible to others.

Several participants reported they were also actively parenting and found the combined responsibilities of work, parenting, and NMOSD high. Others reported comorbid illnesses alongside NMOSD including psychiatric diagnoses, unrelated orthopedic surgeries, or systemic lupus erythematosus. In some cases, a caretaker of a participant of NMOSD was also a caretaker of children.

### Mitigating factors when NMOSD impacts employment

Several participants noted the value of flexible work schedules, working from home, and “understanding managers.” A common phrase was “fortunate to be able to work from home” and “stay at work” despite the NMOSD symptoms, particularly fatigue. Some noted that working from home can still be physically and mentally taxing, and either way, there is “learning a new normal” at work. Self-employment and free-lance work were associated with more control over managing schedules when NMOSD symptoms flared. Examples included taking naps when needed or taking full days off for symptoms or appointments. The understanding of managers and bosses as well as the “ethos of the office” were positive factors in several cases. Several participants reported that maintaining a job after an NMOSD diagnosis preserved a “sense of normalcy” as other aspects of their lives were drastically changed.

Students in higher education programs found that their universities were overall very accommodating and willing to adjust their program enrollment duration and other provisions such as longer times to sit examinations.

Achieving stabilization on a DMT for NMOSD was helpful for several participants over time, as was establishing a longitudinal relationship with an NMO specialist who could provide care. Some participants reported that living nearby an academic medical center with NMO expertise was an advantage. Others noted the value of telehealth to accommodate their schedules without travel.

Many participants found meeting other people with NMOSD to be helpful, supportive, and “calmed fears.” Most participants reported knowing no one else with NMOSD personally, limiting their ability to predict their future experiences or have support from someone familiar with the disease.

### Impact of COVID-19

Alongside COVID-19's impact has been a general trend to allow many employees to work from home. As return to in-person work continues, several participants expressed uncertainty. One participant reported “getting COVID-19 three times last year” while immunosuppressed. For people diagnosed during the pandemic, returning to the office as someone immunosuppressed was associated with significantly increased stress. Some reported comfort with stricter workplace environments for mandatory COVID-19 booster vaccines, required testing for COVID-19 to return to work, and related measures. The return to work was reported as “nerve wracking” given the risks of COVID-19 but also the need to “not make waves during a recession” was reported. One participant stated she could not take a job working with children due to her high concern of getting COVID-19 and other respiratory infections from children at the workplace. Masks were felt to be particularly helpful for the side effects of steroids, including hiding moon facies and obscuring lip biting and other signs of irritability while on steroids.

### Impact of NMOSD on income

Several NMOSD participants reported federal disability assistance. To gain additional income, the need for hourly jobs or hour-by-hour paid tasks such as online surveys, social media related opportunities, freelance writing, and short, work from home jobs was emphasized. This was due to the uncertainty of how NMOSD symptoms would affect work performance the next day. “It is difficult to get a real job,” stated one respondent. The fear of being fired from the job was reported in some cases, especially since some participants needed the money, need the health insurance, and “were barely making ends meet.” One reported there is a risk her partner will lose his job due to the recessionary environment and she has no option to leave her position now.

### Impact of NMOSD on new and future employment and higher education opportunities

Three themes emerged when discussing future opportunities: (1) loss of ambition and reduced prioritization of work with a chronic illness, (2) worry about a future employer being less accommodating of one's needs with NMOSD than the present employer, and (3) need for less physical work, necessitating career changes. Most participants were not presently looking for a job, either confirming that they would be on disability status or reasonably satisfied with their current positions. Several reported the risks of changing jobs since health insurance is tied to their current job. Since their health insurance was working for them for the DMT and visit costs' coverage, they felt “stuck” in their jobs and unable to seek out new work opportunities. There was self-doubt on the ability of participants to succeed in a new job, especially if the current job was comfortable or accommodating to their needs. Among those who switched jobs after an NMO diagnosis, there were mixed results, with some succeeding in the same position and finding it more accommodating, and one returning to her prior employer.

For higher education, goals tended to be dropped or changed. Even though universities were accommodating, participants expressed self-doubt about their ability to complete programs due to their NMOSD symptoms. One participant shared, “I don't feel as ambitious as I was.” Another stated that she accepted she would be in her doctoral program for more years than is usual for her program. Another stated she was “on my way to law school” but visual symptoms made her computing and reading more difficult. She reported she was “not mentally able to do it.” The “workload and stress” were barriers when she needed to “focus on health.”

### Unmet needs related to NMOSD and employment

Several participants reported a need for better community and employer understanding of NMOSD, particularly as distinct from MS. The reference to information found on Google and other online search engines, finding out-of-date information about the near-term mortality of NMOSD, was especially burdensome. Some participants told their employer that they have a condition that is similar to MS, but expressed regret given the stereotypes some people have about aggressive cases of MS, the cognitive component of MS, and the difference in clinical features and some treatments. A more up-to-date, modern, and public information source of information that is useful for employers could be made.

Participants often reported the value of work from home accommodations, for their mobility, toileting, relief of pain and fatigue, driving concerns, and vulnerable immunosuppressed state. There is uncertainty on employer's continuing accommodations for some jobs as COVID-19 changes and “return to the office” mandates occur. Participants who already worked from home and were able to do so throughout the pandemic were more likely to feel optimistic about retention in their current positions. Education on immunosuppression and the risks of infection in group settings was another point of interest for employees who retained in-person employment.

Flexibility of health services was a mitigating factor for several participants, including having MRIs, infusions, or visits, especially before work or on weekends. Telehealth was an advantage. This enabled the concealment of their diagnosis better, avoiding more time off from work, obviating more conversations about their illness, and less emphasis on their inability to be present during work hours.

Identifying opportunities in the workforce for people with college degrees who have difficulty with sensation was identified as an unmet need, including advertisements or curating of job opportunities that are open to people who need assistive technologies to write or type.

There is an opportunity for pharmaceutical companies to ensure bridging of the high-cost DMTs as people with NMOSD change jobs and possibly health insurances. Since insurance for health is combined with employment in the USA, security of a DMT continuing during job transitions is a challenge for higher-frequency and high-cost DMTs especially.

Improving awareness of NMOSD's deficits, at times distinct from a more general concept of physical disability, would help people with NMOSD. Participants felt isolated from the “general disabled community's needs.” Advocacy and support for people within the NMO community was felt to be important, since the symptomatic therapies, unpredictability of future attacks, and immunosuppression posed additional challenges. Meanwhile, familiar concerns, such as a lack of passenger elevator access, poorly accessible lunch facilities, and transportation to work remained present on a case-by-case basis.

## Conclusions

Our study reflects a particular timepoint in the U.S. economy and COVID-19 pandemic and synthesizes the views of 20 people living with NMOSD in the USA. NMOSD is a rare disease affecting an estimated 1 per 1,000,000 people in the USA (12) with three U.S. Food and Drug Administration-approved DMTs and no cure. The participants provided spontaneous and open-ended responses and discussions on the broad topic of employment in NMOSD. The participants' characteristics approximate the general demographic, economic, and geographic composition of the NMOSD population in the USA, coming from 14 states (CA, FL, GA, IL, MA, NJ, ME, MI, MS, MO, OK NH, NV, TX), representing multiple ethnic and racial origins, and demonstrating female predominance (13). Although sampling was by convenience, consecutive, and not population-based, the views of these participants probably reflect the U.S. experience of people with NMOSD in several ways given they derive from a range of settings, employment situations, ages, and disease durations.

Our study was limited by several factors. The coding of the text was done by the investigators themselves and prone to any subconscious biases that may occur. An alternative approach is to use computerized natural language processing as an objective method for determining topical themes as a fully unbiased approach. However, focus group conduct, analysis, and interpretation by a neuroimmunology-trained neurologist may have also facilitated the discussion given the prior awareness of treatments, symptoms, and the U.S. medical system. We did not return transcripts for additional review to participants to minimize participant burden. Our focus groups were conducted online, leading to our need to carefully screen and exclude participants who did not meet stringent criteria of evidence of NMOSD diagnosis. It is possible the dynamic of the conversation and discussion would have changed in person. By contrast, given the risks of group settings for immunosuppressed people during the winter for respiratory infections, the same participants would not have been able to join, especially across the country. Another note is our combination of aquaporin-4 seropositive, MOG seropositive, and double seronegative NMOSD patients. MOG antibody disease was included given the lack of information on the psychosocial impact of MOG antibody disease; however, we note that MOG antibody disease is increasingly recognized as a separate entity from aquaporin-4 antibody seropositive and double seronegative disease. Since NMOSD disease trajectories may reveal differences over the life course depending on presenting autoantibody status, the extrapolation of one participant's experiences to people across disease subgroups—or even within subgroups—should not be presupposed.

The research reported here is meant to be formative. Future studies of a more quantitative nature on the impact of NMOSD on employment remain highly valuable and could benefit from our draft survey instrument, refined by these group discussions, that is meant to address these themes. These focus group comments may also inspire community programs, non-governmental organization (NGO)-based initiatives, and future governmental policies related to work for people with neurological disabilities. This synthesis presents both the challenges and mitigating factors to employment, as told by people with NMOSD. The NMOSD patient's voice is generally lacking in the reported medical literature but provides insights beyond the clinic and thus instructs what clinicians and other stakeholders could do to address real-world issues. While solutions are proposed and suggested for employment in NMOSD, it is likely many more will become available, through new technologies, multidisciplinary disease care, vocational rehabilitation, screening instruments for job loss risk, health systems' sensitivity to health care interference on economic productivity, and improved awareness and education in the community on NMOSD.

## Data availability statement

The original contributions presented in the study are included in the article/Supplementary material, further inquiries can be directed to the corresponding author.

## Ethics statement

The studies involving human participants were reviewed and approved by Mass General Brigham Institutional Review Board. The patients/participants provided their written informed consent to participate in this study.

## Author contributions

FM: drafting, editing, study concept, study supervision, and obtaining funding. CT: data collection, data interpretation, and manuscript editing. Both authors contributed to the article and approved the submitted version.
