# Supplementary material for: Understanding the employment impact of neuromyelitis optica spectrum disorder in the USA: Mixed methods
Source: Front Neurol. 2023 Mar 9;14:1142640. doi: 10.3389/fneur.2023.1142640 (PMC10033531; doi:10.3389/fneur.2023.1142640)
Supplement: Supplementary file 2 [file Data_Sheet_2.PDF]

# NMO and Employment: Survey (English)

Thank you for participating in the international NMO and Employment study. This is the electronic survey. Please feel free to reach out to globalneuroresearch@gmail.com, if you have any questions about the survey.

1) Participant ID number

__________________________________

2) Country

__________________________________

3) Date

__________________________________

4) Who is filling out this survey?

- ◯ The patient
- ◯ Proxy

5) If a proxy, please specify your role (e.g. caregiver, healthcare worker, research coordinator, etc.).

__________________________________

## Participant Demographic Information

6) What is your age (in years)?

__________________________________

7) What is your sex?

- ◯ Male
- ◯ Female
- ◯ Other

8) If other, please specify.

__________________________________

9) What is your race? (Please select all that apply)

- ☐ White
- ☐ Black or African American
- ☐ American Indian or Alaska Native
- ☐ Asian
- ☐ Native Hawaiian or Other Pacific Islander
- ☐ Middle Eastern
- ☐ Some other race

10) If other, please specify.

__________________________________

11) Do you identify as Hispanic or Latino/a?

- ◯ Yes
- ◯ No

12) Which best describes your marital status?

- ◯ Single
- ◯ Married/Partnership
- ◯ Widowed
- ◯ Divorced
- ◯ Separated
- ◯ Prefer not to answer

13) Number of people living in your household:

__________________________________

14) Number of people >18 years old in your household: __________________________________

15) Number of people who earn income in your household: __________________________________
(0 is an acceptable response.)

16) How many years of formal schooling have you attended? __________________________________
(0 is an acceptable response.)

17) Which best describes your highest level of completed education?
- ◯ No school
- ◯ Primary School
- ◯ Secondary school
- ◯ High School/GED
- ◯ Trade School
- ◯ College/Bachelor's Degree
- ◯ Professional Degree

## Pre-NMO Employment History

18) Were you employed at the time of your NMO diagnosis?
- ◯ Yes
- ◯ No

19) What was your job at the time of your first NMO attack? __________________________________

20) Which best describes your employment status at the time of your first NMO attack?
- ◯ Self employed
- ◯ Paid Full Time (40 hours/week)
- ◯ Paid part time
- ◯ Unemployed and looking for work
- ◯ Unemployed and not looking for work
- ◯ Retired
- ◯ Homemaker
- ◯ Disabled
- ◯ Student

21) How many hours per week did you work at/before the time of your first NMO attack? __________________________________
(0 is an acceptable response.)

22) What was your annual income at/before the time of your first NMO attack? (Please answer in your local currency) __________________________________
(0 is an acceptable response.)

## Post-Diagnosis Employment

23) Which best describes your current employment status? (Please choose one)
- ◯ Self Employed
- ◯ Paid Full Time (40 hours/week)
- ◯ Paid part time
- ◯ Unemployed and looking for work
- ◯ Unemployed and not looking for work
- ◯ Retired
- ◯ Homemaker
- ◯ Disabled
- ◯ Student

24) If you are employed, what is your job?

__________________________________

25) How many hours per week do you work outside of your home?

__________________________________
(0 is an acceptable response.)

26) Do you receive health insurance from your employer?
- ◯ Yes
- ◯ No

27) If you responded "yes" to the previous question, was keeping this health insurance a factor in your decision to keep working?
- ◯ Yes
- ◯ No

28) Have you ever lost work due to your diagnosis or symptoms of NMO?
- ◯ Yes
- ◯ No

29) Have you ever reduced your work hours/had your hours reduced due to NMO?
- ◯ Yes
- ◯ No

30) How has your work been affected by your diagnosis and symptoms of NMO?
- ◯ I work less than before
- ◯ I work the same as before
- ◯ I work more than before
- ◯ I no longer work outside the home

31) If you selected "I work less than before," how many hours less per week do you work?

__________________________________

32) If you've stopped working, why did you stop?

__________________________________________

33) Has the COVID-19 pandemic affected your work?
- ◯ Yes
- ◯ No

34) If yes, how so?

__________________________________________

35) How many hours did you work in the last 30 days?

__________________________________
(0 is an acceptable response.)

36) In the last 30 days, how many hours did you miss from work because of problems associated with your NMO? (Include hours missed on sick days/hospitalizations, times you went to work late/left early)

__________________________________
(0 is an acceptable response.)

37) In the last 30 days, how much did your NMO affect your productivity while working? (0=NMO had no effect on my work, 10=NMO completely prevented me from working)

0 5 10

*(Place a mark on the scale above)*

38) If you are a student, how much did NMO disrupt your ability to attend school in the last 30 days?
(0=NMO had no effect on my school, 10=NMO completely prevented me from attending school)

0 5 10

*(Place a mark on the scale above)*

39) In the last 30 days, how much did NMO affect your ability to do your regular daily activities?
(0=NMO had no effect on my activities, 10=NMO completely prevented me from completing activities)

0 5 10

*(Place a mark on the scale above)*

40) In the last 30 days, how much did your NMO symptoms interfere with your normal work? (Both outside the home and housework)

◯ 1-Not at all ◯ 2-Slightly
◯ 3-Moderately ◯ 4-Quite a bit
◯ 5-Extremely

41) What is your annual income? (Please answer in your local currency)

______________________
(0 is an acceptable response.)

42) What is your local currency?

______________________

43) Did your annual income decrease since your diagnosis of NMO?

◯ Yes ◯ No

44) Does your current employer know you have NMO?

◯ Yes ◯ No

45) If your income has decreased since your diagnosis, what percentage of your annual income have you lost due to NMO?
(0%=No lost income due to NMO, 100%=You have lost all of your income due to NMO)

______________________

46) How much of your annual income did you spend on your NMO in the past year? (please provide an amount in your local currency)

______________________

47) Since the onset of your NMO, has anyone regularly helped take care of you without pay? This could be a family member or friend.

◯ Yes ◯ No

48) If you answered "Yes" to the previous question, who regularly helps you manage your NMO?

◯ My spouse/partner ◯ My child/ren
◯ My parent/s ◯ My grandparent/s
◯ My sibling/s ◯ My friend/s
◯ Home nurse/Healthcare worker
◯ Other

49) If other, please specify.

______________________

50) Has this person changed jobs or stopped working at any point to help you manage your NMO?

- ◯ Yes they switched jobs
- ◯ Yes they temporarily stopped working
- ◯ Yes they permanently stopped working
- ◯ Yes they reduced their hours
- ◯ Yes they increased their hours
- ◯ No they have not changed jobs or stopped working

51) If this person changed jobs or their hours, what was the primary reason for this change?

- ◯ Support for hospitalization due to NMO
- ◯ Support for rehabilitation due to NMO
- ◯ Child support/family care
- ◯ Assistance with housekeeping or household chores
- ◯ Other

52) If other, please specify.

__________________________________

53) If this person now works fewer hours per week, how many hours have they lost?

__________________________________

54) If this person now works more hours per week, how many hours have they added?

__________________________________

55) Is there anything else you would like to share about your employment history and NMO?

__________________________________

## NMO Symptoms

56) Do you have fatigue related to NMO?

- ◯ Yes
- ◯ No

57) Has fatigue related to NMO forced you to reduce working hours or stop working altogether?

- ◯ Yes
- ◯ No

58) On a scale of 0-4, how often are you impacted by fatigue due to NMO?

- ◯ 0-Never
- ◯ 1-Rarely
- ◯ 2-Sometimes
- ◯ 3-Often
- ◯ 4-Almost Always

59) On a scale of 0-3, how often have the following problems (little interest or pleasure in doing things) impacted your ability to complete your job duties since your diagnosis of NMO?
(0=Not at all; 1=Several days; 2=More than half the days; 3=Nearly every day)

0 ______________________________ 3

*(Place a mark on the scale above)*

60) On a scale of 0-3, how often have the following problems (feeling down, depressed, or hopeless) impacted your ability to complete your job duties since your diagnosis of NMO?
(0=Not at all; 1=Several days; 2=More than half the days; 3=Nearly every day)

0 ______________________________ 3

*(Place a mark on the scale above)*

61) If you have experienced any of these problems, how difficult have these problems made it for you to do your work, take care of things at home, or get along with other people?
- ◯ Not difficult at all
- ◯ Somewhat difficult
- ◯ Very difficult
- ◯ Extremely difficult

62) Do you experience pain related to your NMO?
- ◯ Yes
- ◯ No

63) Does pain impact your ability to work or complete your job duties?
- ◯ Yes
- ◯ No

64) Did pain impact your decision to work?
- ◯ Yes
- ◯ No

65) Which of the following best represents your average level of pain related to your NMO? (0=No pain; 5=Moderate pain; 10=Worst possible pain)

0 5 10

*(Place a mark on the scale above)*

## History

66) At what age did you experience your first attack of NMO?

__________________________________

67) At what age were you diagnosed with NMO?

__________________________________

68) Have you ever tested positive for the aquaporin-4 (AQP4) antibody?
- ◯ Yes
- ◯ No

69) Have you ever tested positive for the oligodendrocyte glycoprotein (MOG) antibody?
- ◯ Yes
- ◯ No

70) Do you have any of the following conditions?
- ☐ Systemic lupus erythematosus
- ☐ Sjogren's Syndrome
- ☐ Hypertension
- ☐ Diabetes
- ☐ Rheumatoid arthritis
- ☐ Asthma
- ☐ Obesity (BMI>30)
- ☐ Psoriasis
- ☐ Cardiovascular disease
- ☐ Myasthenia Gravis
- ☐ Sarcoidosis
- ☐ Lung disease
- ☐ Gastrointestinal disease
- ☐ Other
- ☐ None

71) If you have lung disease, please specify.

__________________________________

72) If you have gastrointestinal disease, please specify.

__________________________________

73) If other, please specify.

__________________________________

74) Do you smoke?
◯ Yes
◯ No

75) If yes, what do you smoke?
__________________________________

76) Do you drink alcohol?
◯ Yes
◯ No

77) If yes, how many drinks per week?
__________________________________

78) When was your last NMO attack?
__________________________________

79) How many NMO attacks have you had since your diagnosis?
__________________________________

80) Do you have a visual loss/blindness in one or both eyes?
◯ Yes in one eye
◯ Yes in both eyes
◯ No

81) Do you have a history of spinal cord disease/myelitis?
◯ Yes
◯ No

82) Do you need or use unilateral or bilateral walking aids at present?
◯ Yes
◯ No

83) If yes, which one(s)?
☐ Cane
☐ Crutches
☐ Walker
☐ Wheelchair

84) If you receive treatment for NMO, when did you begin treatment? (MM/YY or "Don't Know")
__________________________________

85) If you receive treatment for NMO, what do you take?
__________________________________

86) Other notes or comments, if applicable.
__________________________________________
